# Typhoid Fever

**Published:** 1888-01

**Authors:** 


### *TYPHOID FEVER.*

During the months of July and August, twenty cases of typhoid fever were treated in the Hospital of Notre-Dame, Montreal; seventeen recovered, three died. A resume of the treatment in those cases is as follows: As long as the temperature did not reach 103° Fahrenheit, no antipyretic was exhibited; it was believed that it was then perfectly useless to attempt to lower a temperature that, by itself alone, would not endanger life. When the temperature rose to 103° Fahrenheit or higher, antipyrine was administered, in a fifteen to twenty grain dose, at three o'clock in the afternoon, and a second dose of the same number of grains at five o'clock. The temperature rarely in such cases failed to fall 2° to 4°, and to remain so for six or eight hours. The doses are repeated the day following at the same time if the temperature reaches 103° ; at the same time, quinine is prescribed in accordance with the method of Pecholier, grain twenty to twenty-five a day ; one and a-half to two grains every two hours. Salicylate of soda has not been employed. To combat the thirst, lemonade with a small percentage of hydrochloric acid has been drank. To prevent dryness of the tongue, glycerine has been smeared on ; this has succeeded in the majority of cases, and the patients experienced marked relief from that trouble. In cases of diarrhœa, water impregnated with carbonic disulphide, or, in absence of this, bismuth has been prescribed with the object of rendering the intestinal tract aseptic and, at the same time, of diminishing the frequency of the stools. Finally, when the pulse had a tendency to dicrotism or feebleness, alcoholic stimulants were used: cognac, sherry, champagne, in doses of four, six, eight, ten, twelve, fifteen, sixteen ounces in twenty-four hours, according to circumstances. A liquid diet, easy of digestion, as jelly, eggs, milk, &c. Rest in bed even in cases of walking typhoid. Sleeplessness, if troublesome, has always been treated with chloral (fifteen to twenty grains), given by the mouth or by the rectum when there was no diarrhœa.—*L' Union Medicale du Canada.*

